# The role of drug transporters in the kidney: lessons from tenofovir

**DOI:** 10.3389/fphar.2014.00248

**Published:** 2014-11-11

**Authors:** Darren M. Moss, Megan Neary, Andrew Owen

**Affiliations:** Department of Molecular and Clinical Pharmacology, University of LiverpoolLiverpool, UK

**Keywords:** tenofovir, drug transporters, pharmacokinetics, kidney, toxicity

## Abstract

Tenofovir disoproxil fumarate, the prodrug of nucleotide reverse transcriptase inhibitor tenofovir, shows high efficacy and relatively low toxicity in HIV patients. However, long-term kidney toxicity is now acknowledged as a modest but significant risk for tenofovir-containing regimens, and continuous use of tenofovir in HIV therapy is currently under question by practitioners and researchers. Co-morbidities (hepatitis C, diabetes), low body weight, older age, concomitant administration of potentially nephrotoxic drugs, low CD4 count, and duration of therapy are all risk factors associated with tenofovir-associated tubular dysfunction. Tenofovir is predominantly eliminated via the proximal tubules of the kidney, therefore drug transporters expressed in renal proximal tubule cells are believed to influence tenofovir plasma concentration and toxicity in the kidney. We review here the current evidence that the actions, pharmacogenetics, and drug interactions of drug transporters are relevant factors for tenofovir-associated tubular dysfunction. The use of creatinine and novel biomarkers for kidney damage, and the role that drug transporters play in biomarker disposition, are discussed. The lessons learnt from investigating the role of transporters in tenofovir kidney elimination and toxicity can be utilized for future drug development and clinical management programs.

## INTRODUCTION

Tenofovir, administered as the prodrug tenofovir disoproxil fumarate, is a nucleotide reverse transcriptase inhibitor which is recommended for use in first-line treatment of HIV infection. The drug has many beneficial characteristics, including once-daily dosing, high efficacy, and lack of interaction with cytochrome P450 enzymes (Boffito et al., 2005). Tenofovir shows a favorable safety profile compared to other nucleoside reverse transcriptase inhibitors. However, long-term kidney toxicity is acknowledged as a modest but significant risk for tenofovir-containing regimens (Cooper et al., 2010). It has been observed in a particular clinic that tenofovir-associated nephrotoxicity is the most common single reason for HIV-related referral to specialist renal services, accounting for more than 20% of consultations (Hall et al., 2011). The mechanisms involved in the observed kidney tubular dysfunction are not fully understood, but direct mitochondrial toxicity by tenofovir, interference with normal tubular cell function, or a combination of both have been suggested (Hall et al., 2011). Co-morbidities (hepatitis C, diabetes), low body weight, older age, concomitant administration of potentially nephrotoxic drugs, low CD4 count, and duration of therapy are all risk factors associated with tubular dysfunction (Rodriguez-Novoa et al., 2010). Risk factors may also involve drug transporters expressed in renal proximal tubule cells. Indeed, evidence is emerging that high concentrations of tenofovir in plasma are associated with development of kidney damage, and it is likely that drug transporters play a role in this association (Barditch-Crovo et al., 2001; Rodriguez-Novoa et al., 2009a) as well as in perturbations of the commonly used biomarker, creatinine (Fernandez-Fernandez et al., 2011).

Drug transporters can be divided into two superfamilies; the solute carrier (SLC) superfamily and the ATP binding cassette (ABC) superfamily. It is acknowledged that drug transporters play a significant role in the absorption, distribution, metabolism, elimination (ADME), efficacy, and toxicity of numerous drugs. They are detectable in virtually all tissues, although the precise orientation and function of many transporters are not fully understood (Bleasby et al., 2006). Drug transporters play a key role in controlling the movement of drugs between the blood and the liver (Faber et al., 2003), intestine (Estudante et al., 2013), and kidney (Morrissey et al., 2013). Furthermore, drug transporters are involved in the penetration of drugs into target tissues such as the lymphatic system in antiretroviral treatment (Ford et al., 2004), and also act to protect tissues such as the central nervous system from potentially toxic drugs and xenobiotics (Ballabh et al., 2004). Prior to the licensing of a new drug, the Food and Drug Administration (FDA) and European Medicines Agency (EMA) require that certain tests are performed which determine if a drug is a substrate or inhibitor of a selection of clinically relevant transporters (**Table 1**).

**Table 1:** Recommendations for drug transporter testing as outlined in the EMA Guideline on Investigation of Drug Interactions, July 2012, and the FDA Draft Guidance on Drug Interaction Studies, February 2012.

			Inhibition studies		Substrate studies	
	Transporter	Other name	EMA	FDA	EMA	FDA
efflux	**ABCB1**	**P-gp**	Yes	Yes	Consider	Yes
	**ABCG2**	**BCRP**	Yes	Yes	Consider	Yes
	**ABCB11**	**BSEP**	Preferred	Consider	Consider	Consider
	**ABCCs**	**MRPs**	No	Consider	Consider	Consider
Uptake	**SLC22A6**	**OAT1**	Yes	Yes	Consider	If >25% active renal secretion
	**SLC22A8**	**OAT3**	Yes	Yes	Consider	If >25% active renal secretion
	**SLCO1B1**	**OATP1B1**	Yes	Yes	If >25% clearance is hepatic	If >25% clearance is hepatic or biliary
	**SLCO1B3**	**OATP1B3**	Yes	Yes	If > 25% clearance is hepatic	If >25% clearance is hepatic or biliary
	**SLC22A1**	**OCT1**	Consider	No	Consider	No
	**SLC22A2**	**OCT2**	Yes	Yes	Consider	If >25% active renal secretion
	**SLC47A1**	**MATE1**	Consider	Consider	Consider	Consider
	**SLC47A2**	**MATE2K**	Consider	Consider	Consider	Consider

Tenofovir is predominantly eliminated via the proximal tubules of the kidney, and this review summarizes our current understanding of how kidney transporter polymorphisms and drug interactions may influence tenofovir-associated nephrotoxicity. The implications and knowledge gaps are also described, along with suggestions for future transporter studies. The lessons learnt from investigating the role of transporters in tenofovir kidney elimination and toxicity can be utilized for future drug development and clinical management, which is discussed in this review.

## KIDNEY TRANSPORTERS

The kidney, along with the liver, is a key organ involved in systemic clearance of drugs, with around 32% of currently used drugs in the USA exhibiting significant (>25%) renal elimination (Morrissey et al., 2013). Elimination can occur via glomerular filtration, tubular secretion, or a combination of both pathways. The process of tubular secretion is twofold: (1) the drug requires access to the proximal tubule cells from the blood via the basolateral membrane, and (2) the drug is removed into the luminal fluid via the apical membrane. This process can occur passively, but in many cases drug transporter proteins are involved in facilitating drug movement across membranes and actively transporting drugs against concentration gradients.

Transporters in the kidney are involved in drug–drug interactions, particularly in cases where transport is the main or rate-limiting transmembrane route for a drug. The kidney transporters which are the focus of this review are those where a functional role in drug disposition has been demonstrated or is suspected (**Table 2**) and have been separated into cationic transporters, anionic transporters, transporters with less or unknown specificity in substrate charge, and ABC efflux transporters. It is important to recognize that transporter expression is often not exclusive to a single site in the body, and many have well-defined involvement in tissues other than the kidney (Kis et al., 2010; DeGorter et al., 2012). Several kidney transporters are capable of influencing the elimination of antiretroviral drugs, including tenofovir (Kis et al., 2010). The interactions between tenofovir and kidney transporters are discussed in more detail in a later section.

**Table 2:** Drug transporting proteins expressed in the proximal tubule cells of the kidney.

	Transporter	Other names	Expression	Substrates
Cationic transporters	SLC22A1	OCT1	Basolateral (influx)	**Prostaglandin E2**, **choline**, morphine, tetraethyl ammonium, metformin, aciclovir, lamivudine
	SLC22A2	OCT2	Basolateral (influx)	**Creatinine**, **dopamine**, **histamine**, **prostaglandin E2**, tetraethyl ammonium, pancuronium, MPP, lamivudine
	SLC22A3	OCT3	Basolateral (influx)	**5-HT**, **noradrenaline**, **dopamine**, quinidine, tetraethyl ammonium, MPP
	SLC47A1	MATE1	Apical (efflux)	**Creatinine**, **thiamine**, cimetidine, quinidine, paraquat, cephradine, cephalexin
	SLC47A2	MATE2K	Apical (efflux)	**Creatinine**, **thiamine**, cimetidine, MPP, metformin, aciclovir
Anionic transporters	SLC22A6	OAT1	Basolateral (influx)	**Aminohippuric acid**, **estrone sulfate**, raltegravir, tenofovir, zidovudine
	SLC22A7	OAT2	Basolateral (influx)	**Aminohippuric acid**, **prostaglandin E2**, **estrone sulfate**, paclitaxel, 5-fluorouracil, allopurinol, zidovudine
	SLC22A8	OAT3	Basolateral (influx)	**Aminohippuric acid**, **estrone sulfate**, raltegravir, tenofovir, zidovudine
	SLC22A11	OAT4	Apical (bidirectional)	**Dehydroepiandrosterone**, **estrone sulfate**, **uric acid,** zidovudine
	SLC22A12	URAT1	Apical (bidirectional)	**Uric acid**, **orotic acid**
	SLCO4C1	OATP4C1	Basolateral (influx)	**Steroid conjugates**, **thyroid hormones**, digoxin, ouabain, penicillin
Other transporters	SLC15A1	PEPT1	Apical (influx)	**Oligopeptides**, cyclacillin, valacyclovir, cefadroxil
	SLC15A2	PEPT2	Apical (influx)	**Oligopeptides**, beta-lactam antibiotics, fosinopril
	SLC28A1	CNT1	Apical (efflux)	**Nucleosides**, ribavirin, gemcitabine, zidovudine, zalcitabine
	SLC28A2	CNT2	Apical (efflux)	**Nucleosides**, didanosine, cytidine
	SLC28A3	CNT3	Apical (efflux)	**Nucleosides**, zidovudine, zalcitabine, didanosine
	SLC29A1	ENT1	Basolateral (bidirectional)	**Nucleosides**, ribavirine, 2′,3′-Dideoxyinosine
	SLC29A2	ENT2	Basolateral (bidirectional)	**Nucleosides**, 2′,3′-Dideoxyinosine
ABC transporters	ABCB1	P-gp	Apical (efflux)	**Steroids**, **lipids**, **bilirubin**, **bile acids**, digoxin, doxorubicin, maraviroc, HIV protease inhibitors
	ABCC1	MRP1	Basolateral (efflux)	**Prostaglandins**, **folic acid**, **bilirubin**, anticancer drugs, HIV protease inhibitors
	ABCC2	MRP2	Apical (efflux)	**Bilirubin**, **estradiol glucuronide**, **estrone sulfate**, methotrexate, etoposide, valsartan, HIV protease inhibitors
	ABCC3	MRP3	Basolateral (efflux)	**Bile salts**, **estradiol glucuronide**, anticancer drugs
	ABCC4	MRP4	Apical (efflux)	**Taurocholic acid**, **cAMP**, **cGMP**, **urate**, **prostaglandins**, methotrexate, furosemide
	ABCC6	MRP6	Basolateral (efflux)	Anticancer drugs?
	ABCC10	MRP7	Unknown	**Estradiol glucuronide**, aclitaxel, tariquidar, tenofovir, nevirapine
	ABCG2	BCRP	Apical (efflux)	**Estrone sulfate**, **porphyrins**, anticancer drugs, conjugated organic anions

*Endogenous substrates are in bold. Substrates list is not comprehensive, and examples are given.*

### CATIONIC TRANSPORTERS

SLC22A1, SLC22A2, and SLC22A3 are organic cation transporters expressed on the basolateral membrane of proximal tubule cells. They control the entry of cationic small molecules, including creatinine and numerous drug substrates, into the epithelial cells (Gorboulev et al., 1997; Grundemann et al., 1999; Dresser et al., 2001; Kimura et al., 2002; Urakami et al., 2004; Zhu et al., 2010; Ciarimboli et al., 2012; Tzvetkov et al., 2013). Transporters relevant to this review along with representative drug and endogenous substrates are shown in **Table 2**. Transport is driven by electrochemical potential but is not altered by sodium or proton gradients (Nies et al., 2011). SLC47A1 and SLC47A2, also known as multidrug and toxin extrusion (MATE) transporters, are efflux transporters of cationic substrates (Masuda et al., 2006; Ohta et al., 2006; Chen et al., 2007; Tanihara et al., 2007; Martinez-Guerrero and Wright, 2013). SLC47A1 is highly expressed in the kidney and liver and SLC47A2 is almost exclusively expressed in the kidney, with both showing localization to the apical membrane of proximal tubule cells (Tanihara et al., 2007). Many of the substrates and inhibitors of SLC47 transporters overlap with those of SLC22A1, SLC22A2, and SLC22A3 (Nies et al., 2011). For example, SLC47A1 and SLC47A2 work in cooperation with SLC22A2 to control the concentration of several substrates within proximal tubule cells, such as creatinine (Motohashi and Inui, 2013).

### ANIONIC TRANSPORTERS

SLC22A6, SLC22A7, and SLC222A8 are influx transporters expressed on the basolateral membrane of proximal tubule cells, where they transport small anionic molecules into the cell. SLC22A11 is a related transporter located on the apical membrane and contributes to renal excretion and reabsorption of anionic substrates, as movement of substrates can occur in both directions (Kusuhara et al., 1999; Cha et al., 2000; Kobayashi et al., 2005; Hagos et al., 2007; Moss et al., 2011). Transporters relevant to this review along with representative drug and endogenous substrates are shown in **Table 2**. SLC22A12 is expressed on the apical surface of proximal tubule cells and, in conjunction with SLC22A11, mediates the reabsorption of uric acid from the urine, thereby regulating blood uric acid levels (Enomoto et al., 2002; Vitart et al., 2008). Disruption of SLC22A12 activity through genetic predisposition or drug interactions can cause toxicity, therefore the transporter is considered pharmacologically relevant (Shafiu et al., 2012). The bidirectional transporter SLCO4C1 is highly expressed in the kidney and is located on the apical surface of proximal tubule cells (Bleasby et al., 2006). Substrates of SLCO4C1 include steroid conjugates, thyroid hormones, anti-cancer drugs, and antibiotics (Yamaguchi et al., 2010).

### OTHER TRANSPORTERS

SLC15A1 and SLC15A2 are proton-coupled co-transporters of many diverse peptide and peptidomimetic substrates, but not amino acids (Ganapathy et al., 1995, 1998; Liang et al., 1995; Shu et al., 2001; Daniel and Kottra, 2004; Tramonti et al., 2006). SLC15A1 is expressed on the apical surface of intestinal enterocytes and, to a lesser degree, the apical surface of renal proximal tubule cells, whereas SLC15A2 is expressed predominantly on the apical surface of renal proximal tubule cells. SLC15A2 undertakes the reabsorption of peptide-bound amino nitrogen from the glomerular filtrate, which is important in nitrogen homeostasis (Kamal et al., 2008). Nucleoside transporter proteins are divided into two families; the sodium-dependent, solute carrier family 28 (SLC28), and the equilibrative, solute carrier family 29 (SLC29), where the endogenous substrates are nucleosides or nucleoside-like drugs (Nagai et al., 2006; Endres et al., 2009; Sato et al., 2009; Bhutia et al., 2011; Choi et al., 2014). Again, representative drug and endogenous substrates for these transporters are shown in **Table 2**.

### ABC TRANSPORTERS

Multidrug resistance related proteins (ABCCs) and multidrug resistance protein ABCB1 are members of the ABC superfamily, which can be identified by the presence of a highly conserved ATP binding motif (DeGorter et al., 2012). ABCCs are found in multiple tissues throughout the body, including in relevant ADME tissues such as the small intestine, lymphatic system, liver and kidney, and function in an ATP-dependent process. In the kidney, ABCC2 and ABCC4 are expressed on the apical membrane of proximal tubule cells and efflux anionic substrates such as weakly acidic drugs, glutathione, sulfates, and xenobiotics (DeGorter et al., 2012). ABCC1, ABCC3, and ABCC6 are expressed on the basolateral membrane of proximal tubule cells. ABCC1 does not appear to play a significant role in the absorption or elimination of drugs, but is involved in resistance development of anticancer drugs and in the inflammatory response (Deeley et al., 2006; Bakos and Homolya, 2007). ABCC3 is predominantly expressed in the liver, where it is involved in the regulation of bile salt enterohepatic recirculation, but mRNA is also detectable in numerous other tissues including the kidney (Kool et al., 1999b; Scheffer et al., 2002; Zhou et al., 2008). High *ABCC6* mRNA has been detected in both the liver and kidney (Kool et al., 1999a). However, the exact range of substrates for ABCC6 has not yet been determined, but preliminary investigations suggest that ABCC6 may be involved in the transport of anticancer drugs. ABCC10 is a recent addition to the potentially clinically relevant ABC multidrug resistance proteins, with high mRNA expression found in numerous tissues including the kidney, liver, and intestine (Bleasby et al., 2006). Specificity of expression (i.e., apical or basolateral) is unknown in the proximal tubules, and substrate specificity is limited. However, increasing numbers of drugs, including anticancer and antiretroviral drugs, have been shown to be substrates (Chen et al., 2003; Pushpakom et al., 2011; Liptrott et al., 2012; Sun et al., 2013). ABCB1 is widely distributed in the kidney, liver, small intestine, and brain and is integral for limiting the absorption of potentially toxic xenobiotics into tissues. In the kidney, ABCB1 is expressed on the apical membrane and has broad substrate specificity, although substrates are usually hydrophobic and either neutral or cationic (DeGorter et al., 2012). ABCG2 plays a similar role to ABCB1 in drug disposition, is generally expressed in the same tissues, and contributes to renal excretion of some drugs (Kage et al., 2002; Jani et al., 2009; Beery et al., 2011). Unlike, ABCB1, the substrate preference for ABCG2 includes hydrophilic conjugated organic anions, particularly the sulfate forms. Despite the recent progress made, several drug transporters in the kidney have not been well characterized, and expression levels, locations and substrate affinity remain undetermined.

## TENOFOVIR AND KIDNEY TRANSPORTERS

Tenofovir is predominantly eliminated via the kidney by a combination of glomerular filtration and active tubular secretion. Both influx and efflux transporters are known to influence tenofovir elimination rate, although a complete understanding of the process has not yet been achieved. The efflux transporters ABCC2 (MRP2) and ABCC4 (MRP4) are expressed at the apical surface of proximal tubule cells and actively remove substrates into the renal lumen (Smeets et al., 2004). The level of transport of tenofovir by ABCC2 was found not to be significant (Imaoka et al., 2007; Neumanova et al., 2014). Conversely, ABCC4 has been shown to transport tenofovir and is believed to be the main tenofovir transporter on the apical surface of proximal tubule cells (Kohler et al., 2011). The efflux transporters ABCB1 and ABCG2 are expressed at many membrane barriers in the body, including at the apical surface of proximal tubule cells (Tanigawara, 2000; Woodward et al., 2009). The extent of tenofovir transport by ABCB1 and ABCG2 was assessed *in vitro* and in rodents and found to be not significant (Ray et al., 2006; Neumanova et al., 2014). The Neumanova study also found that the tenofovir prodrug, tenofovir disoproxil fumarate, was a substrate for both transporters. However, it is unlikely that orally administered tenofovir disoproxil fumarate is present at the blood-kidney barrier, as esterase activity rapidly degrades the prodrug in intestinal tissue and plasma following absorption (van Gelder et al., 2002). Nonetheless, ABCB1 and ABCG2 are heavily expressed at the apical surface of the intestinal wall, which is therefore likely to be the major cite where orally administered tenofovir disoproxil fumarate could encounter these transporters. Therefore, it may well be that tenofovir plasma concentrations, and therefore the extent of tenofovir-exposure-associated nephrotoxicity, are influenced by the actions of these transporters on tenofovir disoproxil fumarate absorption. The efflux transporter ABCC10 is known to confer resistance to several anti-cancer drugs (Hopper-Borge et al., 2009; Sun et al., 2013, 2014), and there is growing evidence that it plays a role in tenofovir-associated kidney toxicity. ABCC10 RNA is detectable at high levels in several pharmacologically relevant tissues, including the intestine, liver, brain, and kidney (Bleasby et al., 2006), although protein expression levels, orientation at blood-tissue membrane barriers and substrate specificity are not fully understood. The transport of tenofovir by ABCC1 has been demonstrated *in vitro* (ABCB10-transfected HEK293 cells) and *ex vivo* (ABCC10 siRNA knockdown in CD4^+^ T cells; Pushpakom et al., 2011). However, the potential impact of kidney expression of this transporter *in vivo* has not otherwise been well characterized.

Tenofovir contains a phosphate group with a negative charge at physiological pH, and this gives the drug an affinity for anion-specific influx transporters. Tenofovir is transported by SLC22A6 and, to a lesser extent, SLC22A8 (Uwai et al., 2007). Although affinity of tenofovir for SLC22A6 transporter is greater, SLC22A8 shows higher expression levels in the kidney. As such, this low-affinity high-capacity SLC22A8 transport route may also be important in tenofovir elimination. There remain several kidney-expressed transporters which may be involved in tenofovir-associated nephrotoxicity but which have not been comprehensively assessed for tenofovir transport. The influx transporter SLC22A7 is expressed on the basolateral surface of proximal tubule cells and may work in conjunction with the similar transporters SLC22A6 and SLC22A8 in tenofovir excretion. SLC22A11 is expressed on the apical surface of proximal tubule cells and is able to transport substrates in both directions. The concentrative nucleoside transporters SLC28A1 and SLC28A2 are expressed on the apical surface of proximal tubule cells. Concentrative nucleoside transporters are known to transport the anti-HIV nucleoside analog zidovudine (Hagos and Wolff, 2010) but transport of tenofovir has not been investigated. It is unknown if SLC28A1, SLC28A2, SLC22A7, or SLC22A11 transport tenofovir, and this is certainly worthy of clarification (Hagos and Wolff, 2010).

## TENOFOVIR AND KIDNEY TRANSPORTER PHARMACOGENETICS

It has been proposed that genetic polymorphisms in renal transporters may predispose individuals to have high intracellular tenofovir concentrations, thus increasing the chance of developing tubular toxicity. *ABCC2* polymorphisms have been evaluated, and the haplotype “CATC” [a combination of the polymorphisms at positions –24 (rs717620), 1249 (rs2273697), 3563 (rs8187694), and 3972 (rs3740066) within the *ABCC1* gene] and the allele -24C > T (rs717620) have both been associated with an increased incidence of tenofovir-associated tubular toxicity (Izzedine et al., 2006; Rodriguez-Novoa et al., 2009b). In a study in Japanese HIV^+^ patients, the *ABCC2* –24C > T and 1249G > A polymorphisms were found to be protective for tenofovir-induced kidney toxicity (Nishijima et al., 2012). These observations are difficult to rationalize because tenofovir is not a substrate for ABCC2, which conversely would suggest that ABCC2 activity and expression would not be relevant to tenofovir-associated kidney toxicity *in vivo* (Imaoka et al., 2007; Neumanova et al., 2014). It may be the case that an endogenous substrate for ABCC2 exacerbates the toxicity of tenofovir or competes with tenofovir for transport by ABCC4. Also, the *ABCC2* genotypes may be in linkage disequilibrium with other polymorphisms in genes coding for unidentified factors which exacerbate tenofovir toxicity.

Currently, it is a matter of controversy whether *ABCC4* polymorphisms alter the risk of tenofovir-induced kidney toxicity. A study in HIV^+^ patients found that a 669C > T (rs899494) polymorphism in the *ABCC4* gene was associated with tenofovir-induced kidney toxicity, but this was not found in a subsequent study (Izzedine et al., 2006; Rodriguez-Novoa et al., 2009b). Several additional single nucleotide polymorphisms in *ABCC4* were investigated [559G > T (rs11568658), 912G > T (rs2274407), 951G > T (rs2274406), 969G > A (rs2274405), 1497C > T (rs1557070), 3310T > C (rs11568655), and 3348A > G (rs1751034)] but no associations with tenofovir-induced kidney toxicity were found. The *ABCC4* polymorphism 4131T > C (rs3742106) has been associated with increased concentrations of tenofovir diphosphate (35% higher than homozygotes for the common allele) in human peripheral blood mononuclear cells (PBMCs) 24 h post-dose (Kiser et al., 2008a). The ABCC10 efflux transporter is capable of transporting tenofovir *in vitro* and subsequently polymorphisms of ABCC10 may influence tenofovir disposition. In patients taking tenofovir therapy, two *ABCC10* polymorphisms [526G > A (rs9349256) and 2843T > C (rs2125739)] were associated with kidney toxicity (Pushpakom et al., 2011) but no replication studies have been conducted.

ABCB1 is unlikely to transport tenofovir at the kidney, but the prodrug tenofovir disoproxil fumarate may be influenced by ABCB1 activity at the intestine (as discussed above). Several *ABCB1* polymorphisms [1236C > T (rs1128503), 2677G > T/A (rs2032582), and 3435C > T (rs1045642)] have been analyzed and were found not to be associated with tenofovir-induced kidney toxicity or alteration in tenofovir renal clearance (Izzedine et al., 2006; Rodriguez-Novoa et al., 2009b). Regarding influx transporters, *SLC22A6* polymorphisms 453G > A (rs4149170) and 728G > A (rs11568626) have been analyzed and were found not to be associated with kidney toxicity or alteration in tenofovir renal clearance (Kiser et al., 2008b; Rodriguez-Novoa et al., 2009b).

Pharmacogenetics of relevant drug transporters provides a tool for identifying patients at risk when taking tenofovir. However, pharmacogenetics studies in this context have met with mixed success. Only ABCC2 has shown strong evidence of association with kidney damage phenotypes in patients taking tenofovir. Other associations have been contradicted in further studies, been performed in too few patients to make reliable conclusions or else no replication studies have been attempted. Since non-genetic factors, such as old age, low body weight, co-administered medicines, and co-morbidities are important; it seems likely that transporter genetics will not be fully predictive of the toxicity. Further investigations into the actions of drug transporters may improve our understanding of factors controlling tenofovir disposition and elimination. The pharmacogenetics of the nuclear receptors which control expression of certain transporters, such as the pregnane X receptor and the constitutive androstane receptor, may also be relevant factors, as has been shown for other pharmacological phenotypes involving transporters (Owen et al., 2004; Johnson et al., 2008; Martin et al., 2008; Siccardi et al., 2008; Schipani et al., 2010; Wyen et al., 2011).

## TENOFOVIR AND KIDNEY TRANSPORTER DRUG INTERACTIONS

When co-administered with tenofovir in highly active antiretroviral therapy (HAART), ritonavir-boosted protease inhibitors have been shown to increase tenofovir plasma exposure. An increase in tenofovir AUC of 37 and 32% was observed following co-administration of atazanavir and lopinavir, respectively (Tong et al., 2007). Less substantial increases have been observed for co-administered darunavir (22%), and saquinavir (14%). Ritonavir, and lopinavir inhibit relevant transporters SLC22A8 and ABCC4 *in vitro*, and a transporter-mediated drug interaction at the kidney may explain the elevated tenofovir concentrations when using these drugs (Cihlar et al., 2007). Proteinuria, the presence of an excess of serum protein in the urine, is indicative of kidney functional impairment. The co-administration of protease inhibitors with tenofovir increased the frequency of proteinuria development by sevenfold, compared to tenofovir treatment not containing protease inhibitors (Kelly et al., 2013). This is supported by a further publication that showed use of protease inhibitors to be a predictor of tubular toxicity in tenofovir-containing regiments (Calza et al., 2011). The authors hypothesized that the causes of this association include ritonavir-driven inhibition of enzymes involved in tenofovir elimination from the kidney. However, ritonavir is not known to be involved in affecting metabolism of tenofovir at the kidney, and it seems more likely that ritonavir and other protease inhibitors may inhibit the removal of tenofovir from the kidney proximal tubule cells by inhibiting kidney-expressed transporters, or by preventing tenofovir disoproxil fumarate degradation at the intestine (Tong et al., 2007). Interestingly, a further study by Calza et al. (2013) found that both the development of proteinuria associated with tenofovir use was more pronounced when co-administered with atazanavir, compared to tenofovir co-administered with lopinavir. This data is supported by a further study showing lopinavir to have less severe toxicity-associations compared to other atazanavir, when co-administered with tenofovir (Young et al., 2012). These data suggest that, to reduce the occurance of proteinuria in patients, certain protease inhibitors may be a more suitable addition in a tenofovir-containing regiment.

Other classes of antiretroviral have led to drug interactions with tenofovir. The co-administration of the integrase inhibitor raltegravir with tenofovir disoproxil fumarate resulted in a moderate increase (49%) in tenofovir AUC (Wenning et al., 2008). This interaction may in part be explained by an interaction involving SLC22A6, as raltegravir is capable of inhibiting SLC22A6 *in vitro* (Moss et al., 2011). However, the clinical significance of this interaction is unknown. The use of tenofovir disoproxil fumarate with the nucleoside analog didanosine has been associated with severe side effects, including a reduction in CD4^+^ cell count, pancreatitis, and hyperglycemia. Tenofovir and didanosine are both nephrotoxic and therefore the interaction may result from the additive toxic effects of both drugs. Additionally, tenofovir is capable of increasing didanosine AUC by 44%, which may involve inhibition of SLC22A6-mediated excretion of didanosine via the kidney (Ray et al., 2004). Due to the severity of the drug interaction, co-administration of tenofovir disoproxil fumarate and didanosine is not recommended.

In addition to co-administered antiretrovirals, any other drug which has the potential to compete with tenofovir for kidney excretion via drug transporters may alter tenofovir exposure. In a study using HIV patients, co-administration of the non-steroidal anti-inflammatory drug diclofenac with tenofovir led to a high (14.6%) occurrence of acute kidney injury, compared to tenofovir treatment without diclofenac (0%; Bickel et al., 2013). Diclofenac is an inhibitor of SLC22A6 and ABCC4 and the increased frequency of acute kidney injury in the diclofenac-administered group may be due to inhibition of transporter-associated tenofovir renal excretion (El-Sheikh et al., 2007; Juhasz et al., 2013). However, tenofovir plasma concentrations were not measured in the study and other mechanisms may also be responsible. Further information about drug interactions with tenofovir can be found at the Liverpool drug interactions website (www.HIV-druginteractions.org).

## TENOFOVIR ALAFENAMIDE FUMARATE

A new prodrug of tenofovir, tenofovir alafenamide fumarate, has been developed which is able to target HIV-susceptible CD4^+^ cells by selective intracellular hydrolysis by enzymes expressed within these cells. This has led to a greatly reduced dose of tenofovir being required for effective treatment, as the prodrug is relatively stable in plasma (Markowitz et al., 2014; Sax et al., 2014). Tenofovir alafenamide fumarate is not transported by SLC22A6, meaning that concentrations of drug in the kidney are unlikely to be high (Bam et al., 2014). A lower dose and less propensity for concentrating in the kidney suggest that tenofovir alafenamide fumarate is a potential solution to the issues associated with tenofovir disoproxil fumarate. However, it should be noted that the toxicities associated with tenofovir alafenamide fumarate have not been fully investigates in long-term studies. Furthermore, tenofovir disoproxil fumarate is about to enter the generic drugs market, making it potentially more easily available for widespread distribution in developing countries, and the use of the drug in pre-exposure prophylaxis trials has shown continued success (Bender, 2013). For this to occur successfully, it will still be beneficial for any related renal toxicities to be predictable and preferably avoidable.

## THE EMERGING ROLE OF KIDNEY TRANSPORTERS FOR OTHER DRUGS

Clinically relevant renal drug interactions are rare, but drug transporters are believed to be involved in the majority of reported cases. A well-established inhibitor of anionic transporters is probenecid, which has been used to enhance the activity of penicillin by inhibiting anionic transporters (SLC22A6 and SLC22A8) in the kidney (Robbins et al., 2012). Subsequently, clinical interactions have been observed between probenecid and other drugs, where reduced renal clearance has been observed for acyclovir (↓32%), cefmetazole (↓40%), cidofovir (↓38%), fexofenadine (↓68%), and oseltamivir (↓52%), following probenecid co-administration (Laskin et al., 1982; Ko et al., 1989; Cundy et al., 1995; Hill et al., 2002; Yasui-Furukori et al., 2005). Metformin is a substrate for SLC22A2 and SLC47A1, and these transporters are believed to be involved in the observed reduction in metformin renal clearance when co-administered with cimetidine (↓27%; Somogyi et al., 1987; Tsuda et al., 2009). Digoxin is a substrate for ABCB1, and renal clearance of the drug is reduced when co-administered with ABCB1 inhibitors ritonavir (↓35%) and quinidine (↓34%; Fenster et al., 1980; De Lannoy et al., 1992; Ding et al., 2004).

There are several nephrotoxic drugs, such as didanosine (Cote et al., 2006), cidofovir (Ortiz et al., 2005), cisplatin (Goren et al., 1986) and adefovir (Izzedine et al., 2009), which cause renal failure by accumulating in proximal tubule cells. In these and other cases, targeted inhibition of cellular uptake may reduce nephrotoxicity risks. An example of this strategy is represented by probenecid (an inhibitor of SLC22A6) being used to minimize concentrations of cidofovir in proximal tubule cells (Ho et al., 2000). Prophylaxis with probenecid can be considered in patients receiving cidofovir who have a baseline creatinine serum level of more than 1.5 mg/dL (Choudhury and Ahmed, 2006).

## TRANSPORTERS AND THE COMMONLY USED RENAL BIOMARKER CREATININE

Creatinine is an endogenous waste product of skeletal muscle metabolism and is widely used as a biomarker for renal health. Excretion of creatinine occurs predominantly through glomerular filtration, with proximal tubular secretion accounting for around 15% of total renal clearance. Creatinine is transported into proximal tubule cells by SLC22A7 with a threefold higher affinity than that seen for transport via SLC22A2 and SLC22A3, and efflux into the proximal lumen occurs via SLC47A1 and SLC47A2 by low-affinity high-capacity transport (Urakami et al., 2004; Lepist et al., 2014). Baseline serum creatinine concentration in the blood varies depending on multiple factors, as previously described by Goicoechea et al. (2008). Increase in the serum concentration of creatinine is commonly regarded as an indicator of declining renal health, although serum creatinine concentration has been suggested to poorly represent actual filtration rate (Urakami et al., 2004).

When glomerular filtration rate is low, the serum creatinine concentration and creatinine clearance rate are higher than the actual glomerular filtration rate (Urakami et al., 2004) and this is due to proximal tubule cells secreting creatinine into the tubular lumen. In this circumstance it may be necessary to measure serum creatinine concentrations alongside creatinine clearance to estimate filtration rate in the glomerulus more accurately. Estimated glomerular filtration rate can be calculated through several predictive equations, the most clinically useful being the Cockcroft–Gault and the Modification of Diet in Renal Disease (MDRD) equation (Robertshaw et al., 1989; Estrella and Fine, 2010). Both of these equations are known to have diminished precision at higher glomerular filtration rates (Estrella and Fine, 2010). The site of tenofovir toxicity is believed to be the mitochondria of proximal tubule cells and is achieved by inhibition of mitochondrial DNA polymerase γ (Pushpakom et al., 2011). This toxicity can produce both acute and chronic kidney injury and, less commonly, Fanconi syndrome defined as tubular proteinuria, aminoaciduria, phosphaturia, glycosuria, and bicarbonate wasting (Fernandez-Fernandez et al., 2011; Hall et al., 2011). The effect of tenofovir on creatinine concentration is generally reversible once the tenofovir regimen has ended, but for actual tenofovir-induced kidney tubule dysfunction this is not necessarily the case and therefore the distinction between these scenarios is essential in patients taking tenofovir disoproxil fumarate as part of HAART (Gupta et al., 2014; Solomon et al., 2014). Appropriate screening for abnormal proximal tubule function is necessary throughout a tenofovir regimen and this is achieved through calculating the retinol binding protein to creatinine ratio, a widely used reliable marker for proximal tubule damage (Bernard et al., 1987; Hall et al., 2011; Del Palacio et al., 2012).

Studies investigating the relationship between tenofovir exposure and kidney function have produced mixed results (Hall et al., 2011). Overall, tenofovir is not believed to produce glomerular toxicity (Hall et al., 2011). As creatinine is only excreted by proximal tubule cells to a small degree, a modest decline in estimated glomerular filtration rate may be observed in tubule toxicity. In the case of tenofovir, creatinine is unlikely to be an adequate indicator of renal toxicity and may provide a false positive for reduced glomerular filtration. Further investigation is required in order to elucidate the mechanism of this tenofovir/creatinine interaction.

Multiple drugs have been reported to alter estimated glomerular filtration rate with minimal evidence of actual kidney damage (Berglund et al., 1975; Van Acker et al., 1992; Lepist et al., 2014). The second generation integrase inhibitor dolutegravir and the pharmacological booster cobicistat are two examples with well-characterized mechanisms of creatinine transporter inhibition in the proximal tubule. Cobicistat inhibits SLC47A1 and dolutegravir inhibits SLC22A2, which both transport creatinine through to the proximal lumen (German et al., 2012; Koteff et al., 2013; Lepist et al., 2014).

## EMERGING BIOMARKERS FOR KIDNEY FUNCTION

The contribution of transporter-interaction to the apparent unreliability of creatinine as a biomarker for kidney damage necessitates further research for more appropriate biomarkers. Greater precedence has been given to the development of novel biomarkers with the aim of identifying those that can detect acute kidney injury and progression to chronic kidney damage. To avoid similar issues to those previously discussed with creatinine it is imperative that these biomarkers do not interact with kidney transporters, and this will aid successful intervention before permanent damage to the kidneys occurs. Although no consensus has yet been reached, promising novel biomarkers include cystatin C, asymmetric dimethylarginine (ADMA), neutrophil gelatinase-associated lipocalin, and KIM-1 amongst others (**Table 3**; Han et al., 2002; Herget-Rosenthal et al., 2004; Devarajan, 2008; Estrella and Fine, 2010; Fassett et al., 2011; Schwedhelm and Böger, 2011; de Geus et al., 2012). ADMA has a relatively low molecular weight compared to the other biomarker in **Table 3**, and similarly to creatinine is showing affinity for transporters involved in drug interactions. The biomarkers in **Table 3** with large molecular weights are unlikely to be a substrate for drug transporters. However, transport of albumin via the megalin/cubilin system is the topic of current research, as albumin elevation in plasma has been associated with damage to proximal tubule cells (Dickson et al., 2014).

**Table 3:** Comparison of creatinine with novel biomarkers associated with nephrotoxicity.

Biomarker	Molecular weight (g/mol)	Nephron segment	Kidney transporter interaction	FDA approved^1^
Creatinine	113	Glomerulus	SLC22A2 SLC22A3 SLC47A1 SLC47A2	Yes
ADMA	202.5	Non-specific	SLC22A2 SLC47A1	No
TFF3	6600	Glomerulus Proximal tubule	No	No
β2-Microglobulin	11,800	Glomerulus and Proximal tubule	No	No
Cystatin C	13,300	Glomerulus and proximal tubule	No	No
NGAL	25,000	Proximal tubule and Distal tubule	No	No
KIM-1	30,000	Proximal tubule	No	No
Clusterin	75–80,000	Proximal tubule and distal tubule	No	No

*^1^FDA approval defined as approved for use in clinical setting. ADMA, asymmetric dimethylarginine; KIM-1, kidney injury molecule 1; NGAL, neutrophil gelatinase associated lipocalin; TFF3, trefoil factor 3.*

## DATA FOR OTHER TRANSPORTERS WITH PUTATIVE RENAL IMPORTANCE

As our understanding of drug transporters improves, it is becoming clear that transporters can play an important role in disease development. Experiments with transgenic mice have shown that genetic knockdown of transporters can cause numerous kidney-related morbidities, developmental abnormalities, and even death (**Table 4**). Genetic associations with disease traits (in the absence of drugs) can also be useful for defining mechanisms. The genetics of hyperuricemia and gout is known to involve transporters expressed in the proximal tubule cells. In 2002, genetic variants in SLC22A12 were found to predict occurrence of gout, and this association was joined by further transporters in 2007 (SLC2A9), 2008 (ABCG2, SLC17A3, SLC17A1, SLC16A9, SLC22A11), and 2011 (SLC2A12; Reginato et al., 2012). Understanding that multiple transporters are usually involved in the movement of a drug through the proximal tubule, it can be misleading or even counterproductive to focus on individual transporters in order to discover the “major” players in the elimination of the drug for future pharmacogenetic and interaction studies. There is limited understanding of how kidney transporter expression and activity differ between men and women (Morris et al., 2003), and in special populations, such as in specific disease groups (Lalande et al., 2014), pediatrics (Shen et al., 2001) and geriatrics, and this area requires further investigation.

**Table 4:** The effects of genetic knockdown of kidney transporters in transgenic mice.

Transporter	Other names	Effects of genetic knockdown of transporter	Reference
Abca1	Abc1	Devoid of high-density lipoprotein cholesterol, reduction in serum cholesterol and membranoproliferative glomerulonephritis.	Christiansen-Weber et al. (2000)
Slc13a1	NaSi-1	Serum sulfate concentration reduced by 75%. Growth retardation and reduced fertility observed.	Dawson et al. (2003)
Slc14a2	UT-A	Deletion of UT-A1/UT-A3 resulted in polyuria and a severe urine concentrating defect.	Fenton et al. (2004)
Slc15a2	Pept2	Twofold increase in renal glycylsarcosine clearance resulting in lower systemic concentrations.	Ocheltree et al. (2005)
Slc16a2	Mct8	General hyperthyroid state of the kidneys.	Trajkovic-Arsic et al. (2010)
Slc22a12	URAT1	Decreased reabsorption of urate.	Eraly et al. (2008)
Slc22a1	Oct1	Combined knockout of Slc22a1 and Slc22a2 abolished renal secretion of organic cation tetraethyl ammonium.	Jonker et al. (2003)
Slc22a2	Oct2	Combined knockout of Slc22a1 and Slc22a2 abolished renal secretion of tetraethyl ammonium.	Jonker et al. (2003)
Slc22a6	Oat1	Profound decrease in renal excretion of organic anions (e.g., para-aminohippurate).	Eraly et al. (2006)
Slc22a8	Oat3	Decreased secretion of urate.	Eraly et al. (2008)
SLC26A1	Sat1	Hyperoxaluria with hyperoxalemia, nephrocalcinosis, and calcium oxalate stones in renal tubules and bladder.	Dawson et al. (2010)
Slc26a4	Pendrin	Acidic urine and increased urine calcium excretion.	Barone et al. (2012)
Slc26a6	Pat1	Increased renal succinate uptake, hyperoxaluria, and hypcitraturia.	Ohana et al. (2013)
Slc26a7	SUT2	Distal renal tubular acidosis manifested by metabolic acidosis and alkaline urine pH.	Xu et al. (2009)
Slc2a9	Glut9	Moderate hyperuricemia, severe hyperuricosuria, and an early onset nephropathy.	Preitner et al. (2009)
Slc34a1	Npt2b	Npt2b(–/–) lethal and Npt2b(+/–) showed hypophosphatemia and low urinary P (i) excretion.	Ohi et al. (2011)
Slc42a3	Rhcg	Urinary ammonia excretion lower and more susceptible to metabolic acidosis.	Lee et al. (2009)
Slc4a8	ENaC	Disrupted fluid homeostasis.	Leviel et al. (2010)
Slc5a12	SMCT2	Combined knockout of SLC5A8 and SLC5A12 (c/ebpdelta–/–mice) results in marked increase in urinary excretion of lactate and urate.	Thangaraju et al. (2006)
Slc5a2	Sglt2	Glucosuria, polyuria, and increased food and fluid intake.	Vallon et al. (2011)
Slc5a8	SMCT	Combined knockout of SLC5A8 and SLC5A12 (c/ebpdelta–/–mice) results in marked increase in urinary excretion of lactate and urate.	Thangaraju et al. (2006)
Slc6a18	Xtrp2	Higher glycine excretion and higher systolic blood pressure.	Quan et al. (2004)
Slc7a8	LAT2	Increased urinary loss of small neutral amino acids.	Braun et al. (2011)
Slc7a9	BAT1	Develop a cystinuria-like phenotype with hyperexcretion of cystine and dibasic amino acids.	Feliubadalo et al. (2003)
Slc9a3	NHE3	Diarrhea and blood acidosis. HCO3- and fluid absorption are reduced in proximal convoluted tubules.	Schultheis et al. (1998)

## CONCLUSION: PERSPECTIVES ON TRANSPORTERS IN THE KIDNEY

**FIGURE 1:**
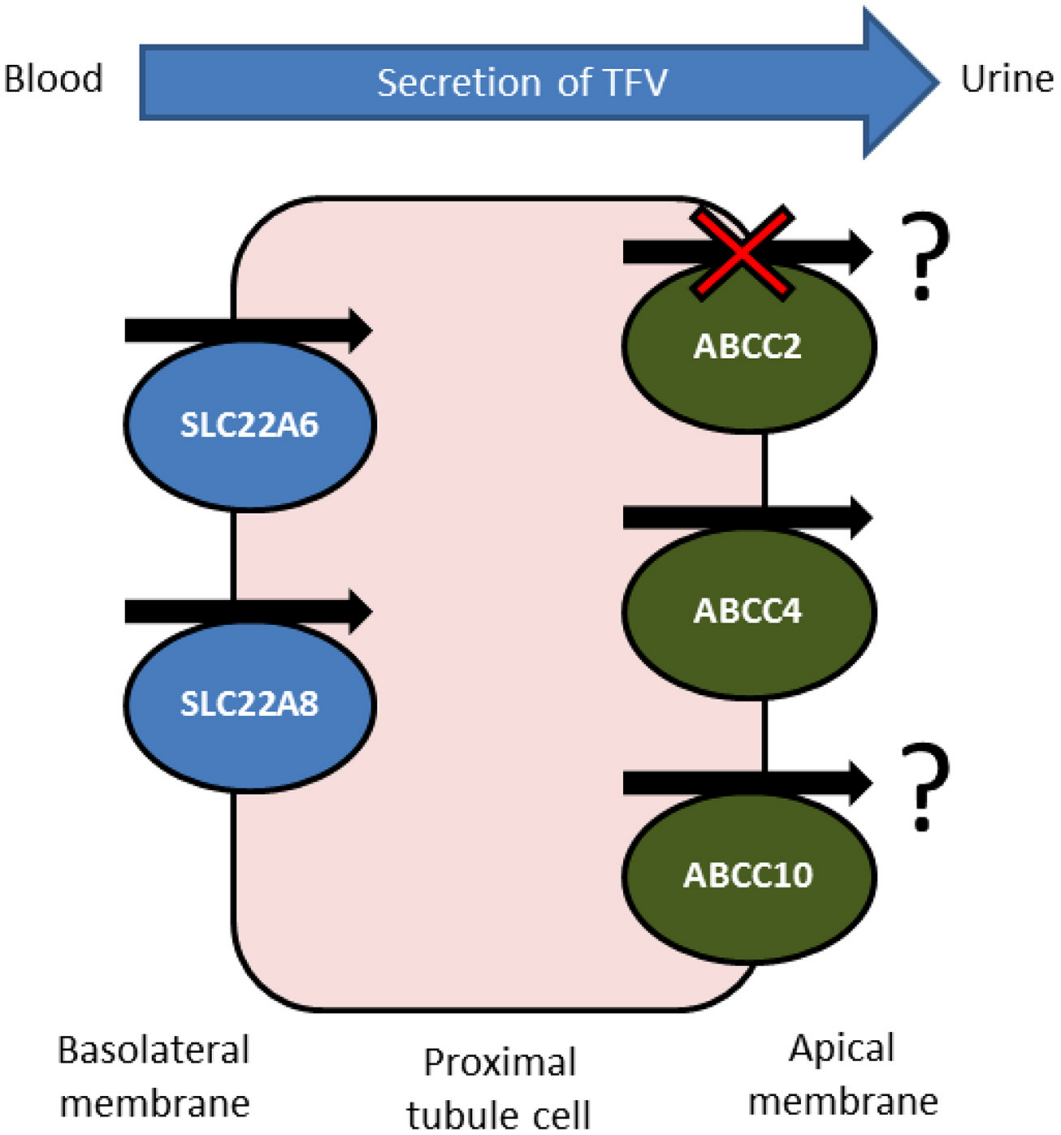
**Confirmed and potential transporters involved in active tubular secretion of tenofovir into urine.** Tenofovir is removed from the circulating blood and enters the proximal tubule cells by the actions of basolaterally expressed SLC22A6 and, to a lesser extent, SLC22A8. Tenofovir is then removed into the tubular lumen by apically expressed ABCC4. ABCC2 does not transport tenofovir *in vitro* but pharacogenetics suggests ABCC2 has a role in tenofovir-induced renal toxicity. The orientation of ABCC10 in proximal tubule cells is unknown, but *in vitro* and pharmacogenetic data suggest that expression may be localized to the apical membrane, facilitating tenofovir secretion.

Despite showing a favorable toxicity profile in initial treatment, the long-term use of tenofovir disoproxil fumarate in HIV therapy is currently under question by practitioners and researchers (Fernandez-Fernandez et al., 2011). Large-scale and long-term studies are continuing to appear which suggest an association between tenofovir use and kidney damage. Despite this, tenofovir is included in first-line therapy for both treatment naive and experienced patients as it is very effective at reducing and controlling HIV replication in patients. Because of this, and due to the life-long nature of antiretroviral therapy, it is essential that a reliable strategy be developed to detect and preferably avoid tenofovir-associated kidney toxicity. It is clear from the summarized evidence that tenofovir plasma concentrations are linked to renal toxicity, and it is also clear that drug transporters, particularly those expressed in the kidney, are able to influence the clearance rate of tenofovir (**Figure 1**) and also interfere with the utility of creatinine clearance as a biomarker.

When looked at more broadly, for the majority of drugs the potential for clinically relevant renal transporter-mediated drug interactions is low, and reported cases are limited. Renal excretion of drugs may be achieved by glomerular filtration as well as tubular secretion, and transporters are only likely to be influential in drug elimination when tubular secretion is the major pathway. Additionally, transporters in the kidney often show overlapping substrate affinity (see **Table 2**) and therefore the inhibition of a single transporter may not produce significant alterations in drug elimination *in vivo*. However, in certain cases the actions of transporters in the kidney can have clinical implications, as emphasized with tenofovir.

Despite decades of research into drug transporters, the recommendations for drug interaction studies provided by the FDA and EMA include testing strategies for only a small fraction of the total expressed transporters in the human body (**Table 1**) and it is unknown whether transporter-associated drug interactions in the kidney will obtain the same relevance as seen with drug metabolizing enzymes and transporters in the intestine and liver. As the investigations into tenofovir elimination have emphasized, determination of the actions of individual transporters in drug elimination from the kidney, even when found to be relevant *in vitro*, often may not be clinically implementable, as drugs are often substrates for several transporters. Indeed, multiple transporters and metabolism enzymes, as well as other biological and drug-specific factors, work in concert to determine the overall disposition of a drug. This should be taken into consideration in future drug development strategies with the use of improved *in vitro* methodologies and the introduction of predictive physiologically based in silico modeling.

## Conflict of Interest Statement

The authors declare that the research was conducted in the absence of any commercial or financial relationships that could be construed as a potential conflict of interest.
