# Corrigendum: The role of drug transporters in the kidney: lessons from tenofovir

**DOI:** 10.3389/fphar.2015.00018

**Published:** 2015-02-09

**Authors:** Darren M. Moss

**Affiliations:** Department of Molecular and Clinical Pharmacology, University of LiverpoolLiverpool, UK

**Keywords:** tenofovir, HIV-1, drug transporters, kidney, toxicity

The authors wish to correct a mistake on the review “The role of drug transporters in the kidney: lessons from tenofovir” regarding the details of a particular ABCC2 polymorphism [−24C > T (rs717620)]. In the Section “Tenofovir and kidney transporter pharmacogenetics” on page five of the article, the second line on the second column should state the ABCC2 polymorphism as −24C > T, rather than −24T > C. Additionally, the sentence beginning on page five, line four of the second column should be changed from: “In a study in Japanese HIV+ patients, the *ABCC2* −24C > T and1249G > A polymorphisms were found to be protective for tenofovir-induced kidney toxicity (Nishijima et al., 2012).” and instead read: “In a study in Japanese HIV+ patients, the *ABCC2* −24T > C and 1249G > A polymorphisms were found to be associated with the increased occurrence of tenofovir-induced kidney toxicity (Nishijima et al., 2012).”

## Conflict of interest statement

The author declares that the research was conducted in the absence of any commercial or financial relationships that could be construed as a potential conflict of interest.
